# Multivesicular release favors short term synaptic depression in hippocampal autapses

**DOI:** 10.3389/fncel.2023.1057242

**Published:** 2023-05-17

**Authors:** Pablo Martínez San Segundo, Beatrice Terni, Artur Llobet

**Affiliations:** ^1^Laboratory of Neurobiology, Department of Pathology and Experimental Therapy, Institute of Neurosciences, University of Barcelona, Barcelona, Spain; ^2^Bellvitge Biomedical Research Institute (IDIBELL), Barcelona, Spain

**Keywords:** presynaptic terminal, synaptic vesicle, neurotransmitter release, short-term synaptic depression, autapse

## Abstract

Presynaptic terminals of the central nervous system can support univesicular and multivesicular synchronous release of neurotransmitters, however, the functional implications of the prevalence of one mechanism over the other are yet unresolved. Here, we took advantage of the expression of SF-iGluSnFR.S72A in the astrocytic feeder layer of autaptic hippocampal neuronal cultures to associate the liberation of glutamate to excitatory postsynaptic currents. The presence of the glutamate sensor in glial cells avoided any interference with the function of endogenous postsynaptic receptors. It was possible to optically detect changes in neurotransmitter release probability, which was heterogeneous among synaptic boutons studied. For each neuron investigated, the liberation of neurotransmitters occurred through a predominant mechanism. The prevalence of multivesicular over univesicular release increased synaptic strength and enhanced short-term synaptic depression. These results show that the preference of hippocampal boutons to synchronously release one or more vesicles determines the strength and low pass filtering properties of the synapses established.

## Introduction

Presynaptic terminals exhibit different modalities of neurotransmitter release to support the correct process of information. The exocytosis of synaptic vesicles can occur immediately upon the arrival of an action potential, with a certain delay, or, in a totally unrelated manner, defining synchronous, asynchronous and spontaneous neurotransmitter release, respectively (Kaeser and Regehr, 2014). Synaptic strength can thus be fine-tuned by balancing the contribution of these three different neurotransmitter release modes. There are, however, mechanisms that allow to gain control of synaptic efficacy without affecting the temporal association between stimuli and responses. An example is through modification of the efficiency of synchronous neurotransmitter release. The number of vesicles undergoing exocytosis upon the arrival of a single action potential can range from one, termed univesicular release (UVR), to more than one, known as multivesicular release (MVR). It was originally assumed that UVR was predominant in the majority of synapses and that MVR occurred in particular types of neurons, as for example those forming ribbon synapses (Glowatzki and Fuchs, 2002). However, data obtained in neurons establishing small synapses, such as hippocampal (Tong and Jahr, 1994), striatal (Higley et al., 2009) or cortical (Huang et al., 2010), revealed the widespread presence of MVR throughout the brain. This evidence raised the possibility that the expression of UVR or MVR is an inherent property of axon terminals, relevant to the determination and regulation of synaptic strength (Rudolph et al., 2015).

In autaptic hippocampal neuronal cultures, where a single neuron establishes reciprocal synapses, there are presynaptic terminals of low and high release probability along the axon (Murthy et al., 1997). Yet the molecular mechanisms setting the bases for the observed skewed distribution of neurotransmitter release probability remain to be identified. A possible origin attributable to culture conditions is unlikely since similar variability is observed in the nervous system, as for example in a population of CA3 hippocampal neurons (Éltes et al., 2017). The cause is a variation in voltage gated calcium channel composition, however, other factors such as the expression levels of munc 13-1 (Holderith et al., 2021) could also be relevant. Whether or how the balance between UVR and MVR is determinant for synaptic heterogeneity remains unexplored. By taking advantage of the reductionistic scenario provided by hippocampal autaptic cultures combined to the use of SF-iGluSnFR.S72A (Marvin et al., 2018), we show that UVR and MVR are normally present in synaptic boutons found along a single axon and are important to short-term synaptic plasticity.

## Materials and methods

### Molecular biology

pAAV.hSynapsin.SF-iGluSnFR.S72A was obtained from Addgene (RRID:Addgene_106176). The coding sequence of SF-iGluSnFR.S72A was cloned into pWPXL (RRID:Addgene_12257) by replacing EGFP using BamHI and NdeI. For lentivirus production, HEK 293T cells were transfected using calcium phosphate following methods described by Didier Trono (http://tronolab.epfl.ch/lentivectors) with pMD2.G (RRID:Addgene_12259), pCMVR8.74 (RRID:Addgene_22036), and pWPXL containing SF-iGluSnFR.S72A. Two days later, culture medium containing lentiviral particles was collected in 3 rounds at 8 h intervals, kept at 4°C, and centrifuged at 500 × g. Supernatants were distributed in aliquots and stored at −80°C.

### Cell culture

Experimental procedures were approved by the Department of Environment from Generalitat de Catalunya. Microcultures of autaptic hippocampal neurons were established using P0 Sprague-Dawley rats. Cortical primary astrocytes were used as a glial feeder layer. The protocol was divided in two-stages (Burgalossi et al., 2012). The first stage of the procedure consisted in the establishment of a primary culture of astrocytes, which was maintained for ~2 weeks. Upon confluency, flasks were trypsinized and astrocytes were seeded at 5,000 cells/mL on 15 mm coverslips containing collagen microdrots of 100–400 μm diameter. Collagen was prepared following methods described elsewhere (Perez-Gonzalez et al., 2008). Culture medium was DMEM/GlutaMAX (Gibco, cat. N°: 31966-021, Paisley, UK) containing 10% heat inactivated FBS (Biological Industries, cat. N°: 04-007-1A Beit HaEmek, Israel), 0.1% MITO + serum extender (Corning, cat. N°: 355006, Glendale, AZ, USA), and 0.2% penicillin/streptomycin. Astrocytes showed a growth limited to microislands after 3–5 days *in vitro* (D.I.V.). Transduction with lentiviruses was carried out by a 1:2 overnight incubation of the viral stock and fluorescence emitted by SF-iGluSnFR.S72A was evident 2 days after infection. The astrocytic feeder layer was thus ready to support the growth of hippocampal neurons, as well as, to detect glutamate release. In the second stage, two hippocampi were dissected out from a new-born rat pup (Sprague-Dawley) and transferred into a 1.5 mL centrifuge tube containing 0.5 mL of papain enzyme solution (Worthington, cat N°: LK003178, Lakewood, NJ, USA). Tissues were digested for 45 min at 37°C in a heating block with gentle shaking (450 r.p.m.), followed by mechanical dissociation. The enzymatic activity was deactivated by carefully discarding the supernatant and adding prewarmed stop solution (DMEM/F12 [1:1] supplemented with 10% heat inactivated FBS and 2.5 mg/ml albumin) for 15 min at 37°C. The hippocampi were carefully triturated by pipetting 10 times with a 1 mL tip. The sediment was let back to the bottom of the tube for ~2 min and the supernatant was transferred to a new centrifuge tube with NBA solution, which contained prewarmed Neurobasal A (Gibco, cat. N°: 10888022, Paisley, UK) supplemented with 2% B27 (Gibco, cat. N°: 17504044, Grand Island, NY, USA) and 1% GlutaMAX (Gibco, cat. N°: 35050061, Grand Island, NY, USA) at 37°C. The cell suspension was carefully mixed, and the cell density was counted in a Neubauer chamber. Dissociated neurons were added to the NBA solution to reach a concentration of 3000 cells/mL. Neurons were seeded on astrocytic microislands expressing SF-iGluSnFR.S72A, by exchanging the glial culture media with the suspension of hippocampal neurons. Recordings of synaptic responses were obtained after 18 days *in vitro* (D.I.V.).

### Electrophysiology and imaging

All experiments were performed in the whole-cell configuration of the patch-clamp mode using neurons microcultured for 18–22 D.I.V. Typical resistances of pipettes used for recordings were ~5 MΩ when filled with internal solution, which contained (in mM): 130 K-gluconate, 4 MgCl_2_, 1 EGTA, 10 HEPES, 3 Na_2_ATP, 1 NaGTP, pH 7.2, 290 mOsm/kg. The composition of the external solution was (in mM): 130 NaCl, 5 KCl, 2 MgCl_2_, 10 HEPES-hemisodium salt, and 10 glucose, pH 7.4. The final 2 mM CaCl_2_ concentration was always achieved by dilution from a 1 M stock. In the indicated experiments SrCl_2_ substituted equimolarly CaCl_2_. All salts were from Sigma-Aldrich (St. Louis, MO, USA). Before the addition of glucose and CaCl_2_, the osmolality of the external solution was adjusted to 290 mOsm/kg. Experiments were performed at room temperature (23°C). Recordings were made using an Axopatch-1D patch-clamp amplifier (Molecular Devices, San Jose, CA, USA) under the control of an ITC-18 board (Instrutech, Port Washington, NY, USA) driven by mafPC (courtesy of M. A. Xu-Friedman, University at Buffalo, NY). Neurons were clamped at −70 mV and stimulated by a 1–2 ms depolarization step that drove membrane potential to 0 mV. The presence of functional autaptic synapses was identified by the generation of excitatory postsynaptic currents (EPSCs) as previously described (Perez-Gonzalez et al., 2008).

Coverslips containing neuronal microcultures were mounted on an RC-25 imaging chamber (Warner Instruments, Holliston, MA, USA) and placed in an inverted Olympus IX-71 microscope to visualize glutamate release using SF-iGluSnFR.S72A. Cells were illuminated with blue light, using a 488/20 nm excitation filter. Fluorescence was acquired using q505LP dichroic and HQ535/50 nm emission filters (Chroma Technology Corp., Bellows Falls, VT, USA). Images were collected through a Plan Apo 60 × , 1.45 NA (Olympus, Tokyo, Japan) objective and visualized using an Orca Flash 4.0 camera (Hamamatsu, Shizuoka, Japan) controlled by Hokawo software (Hamamatsu). Images from a 512 × 512 pixels region were acquired at 100 Hz. A TTL pulse generated by mafPC software controlled the exposure time. Timing of light illumination was adjusted via a shutter (Uniblitz, Rochester, NY, USA) to minimize photobleaching. A TTL pulse opened and closed the shutter at the beginning and end of each recording episode.

### Immunocytochemistry

Coverslips containing representative autaptic hippocampal microcultures were fixed in 4% paraformaldehyde and processed for immunocytochemistry. To quantify the number of excitatory synapses present, samples were incubated overnight at 4°C with monoclonal anti-PSD-95 primary antibody (1:1,000, Synaptic Systems, 124 011), polyclonal anti-vGlut-1 primary antibody (1:1,000, Synaptic Systems, 135 302) and DAPI (1:10,000, Invitrogen, D3571). Neuronal morphology was observed using anti-calretinin antibody (1:1,000, Swant, CR 7697) and astrocytes in the glial feeder layer were characterized using anti-S100 (1:1,000, Abcam, 4066). Appropriate secondary antibodies labeled with Alexa Fluor 488 and Alexa Fluor 555 were used for fluorescent staining. Cells were visualized in a Zeiss LSM 880 confocal microscope (Carl Zeiss AG, Oberkochen, Germany). Optical sections were acquired using a 63X oil immersion objective PL-APO (1.4 N.A.).

### Analysis

Analysis was performed with custom-made macros written in Igor Pro software version 8.0 (Wavemetrics, Lake Oswego, OR). Changes in SF-iGluSnFR.S72A fluorescence were reported as relative changes of basal fluorescence, measured as (F-F_0_)/F_0_ in selected regions of interest (ROIs). The selection of 1.3 × 1.3 μm ROIs in the astrocytic membrane detecting glutamate release was carried out by visual identification in difference movies (|F-F_0_|) followed by the application of a gaussian blur filter. Basal SF-iGluSnFR.S72A fluorescence (F_0_) was defined as the average fluorescence obtained in 95 images before stimulation. Glutamate release was circumscribed to restricted regions of the astrocytic membrane. Non-responding areas were used to calculate background fluorescence. Increases in ΔF/F observed for individual train stimuli were quantified after subtracting the decaying phase of the previous event. The representative response of a given neuron was obtained by averaging the responses of all ROIs identified.

The co-localization of the presynaptic marker vGlut-1 with postsynaptic PSD95 puncta in neurons processed by immunocytochemistry was carried out using the colocalization plugin of ImageJ. A punctum was considered as a putative synapse if it spanned from three to five consecutive confocal Z-sections (0.33 μm optical thickness), had an apparent diameter ranging from 0.3 to 1 μm and was co-stained with vGlut-1 and PSD-95. Synaptic density was calculated dividing the number of puncta identified by the surface of the analyzed area.

### Statistics

Data from averages were always expressed as mean ± s.e.m. For statistical analysis, comparisons between two groups were carried out using paired and unpaired two-tailed Student's *t* test.

## Results

### Visualization of glutamate release in autaptic neuronal cultures

**Figure 1:**
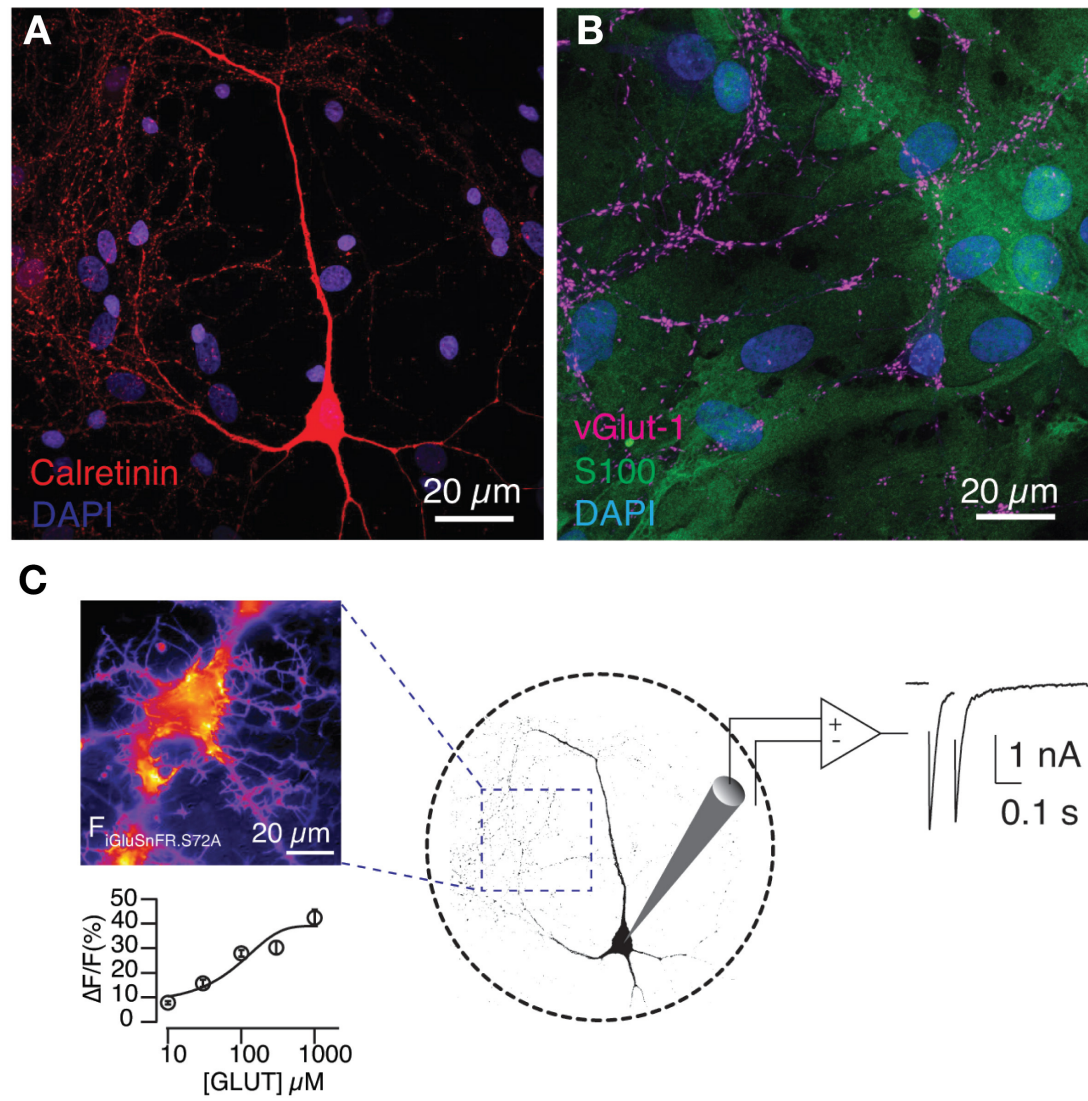
Expression of SF-iGluSnFR.S72A in the astrocytic feeder of autaptic hippocampal cultures detects glutamate release. **(A)** Image of a hippocampal neuron grown in microculture. The nuclei of the glial feeder layer are revealed by DAPI and the morphology of the neuron is visualized by calretinin staining. **(B)** Astrocytes, which contain S100 in their cytoplasm, support the growth of glutamatergic neurons, characterized by the presence of the vesicular glutamate transporter vGlut-1. **(C)** The image on the left was obtained during a titration experiment and shows the increase in fluorescence (a.u.) experimented by an astrocyte in response to the local application of 300 μM glutamate during 0.5 s. Fluorescence responses reported the extracellular [glutamate] with a Kd of 245 ± 4 μM. Circles indicate mean ± s.e.m. (*n* = 12). Right, the experimental configuration allowed the association of SF-iGluSnFR.S72A fluorescence changes to autaptic currents.

Autaptic cultures of hippocampal neurons provide a unique experimental platform to investigate the molecular mechanisms of synaptic transmission. Using a single electrode it is possible to record the excitatory postsynaptic current (EPSC) generated in response to stimulation and evaluate short-term synaptic plasticity (Bekkers, 2020). Here, we sought to maximize the experimental possibilities of this type of preparation to evaluate the implication of MVR and UVR on synaptic responses. Functional autapses of hippocampal neurons can only be achieved in the presence of a glial feeder layer (Figures 1A, B), which is a key difference to neurons of the peripheral nervous system, i.e. the superior cervical ganglion (Nägler et al., 2001; Perez-Gonzalez et al., 2008). Although astrocytes do not provide *in vitro* the characteristic synaptic ensheathment observed *in vivo* (Perea et al., 2009), we evaluated if the existing spatial organization allowed the detection of glutamate leaking out of the synapse. The advantage of expressing the low-affinity sensor SF-iGluSnFR.S72A in astrocytes is the null interference with endogenous glutamate receptors and therefore, this experimental configuration ruled out any modification of neuronal properties caused by exogenous gene expression.

**Figure 2:**
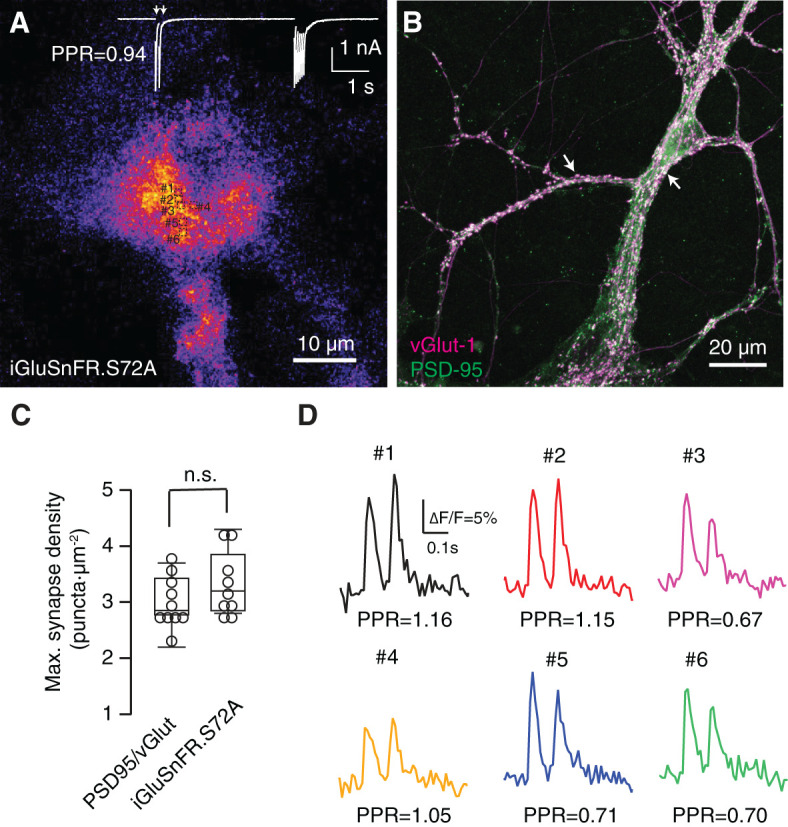
Functional identification of synaptic responses. **(A)** Average of 95 images acquired at 100 Hz displaying fluorescence increases of SF-iGluSnFR.S72A during the application of stimuli. In this example, excitatory postsynaptic currents (EPSCs) were obtained in response to the application of two pulses at an interval of 100 ms followed by a train of 14 stimuli delivered at 20 Hz (white trace). **(B)** Density of autaptic synapses revealed by co-staining with the presynaptyic and postsynaptyic markers vGlut-1 and PSD-95, respectively (arrows). **(A, B)** Show two different cultures. **(C)** Maximum synaptic density estimated by quantifying the co-localization of PSD-95 and vGlut-1 (*n* = 10) and the number of SF-iGluSnFR.S72A fluorescent puncta (*n* = 9) in neuronal and astrocytic regions, respectively. Comparisons were established using unpaired Student's *t*-test. **(D)** Increases in SF-iGluSnFR.S72A fluorescence obtained in the ROIs indicated in **(A)** in response to paired pulse stimulation (arrows). The paired pulse ratio (PPR = Peak_2_/Peak_1_ ΔF/F) obtained for each puncta can be compared to PPR measured electrophysiologically in **(A)**, which reflects the short-term synaptic plasticity of autapses present in the culture. Individual traces illustrate single trial events.

SF-iGluSnFR.S72A was fully functional in the astrocytic membrane and experimented fluorescence changes that were proportional to the extracellular concentration of glutamate (Figure 1C). Titration experiments revealed a Kd of 245 ± 4 μM (*n* = 12), in agreement with previous results (Marvin et al., 2018). Although such low affinity could potentially pose a limit to sense basal neurotransmission, SF-iGluSnFR.S72A was indeed capable of detecting glutamate release from autaptic contacts. Astrocytic membrane regions showed an increase in fluorescence that coincided with stimulation (Figure 2A). Responses were local and circumscribed to defined areas of the membrane. SF-iGluSnFR.S72A did not support a wide detection of neurotransmitter release and only those synapses located near astrocytes were presumably detected. An estimate of the minimal distance required to generate a response could be obtained using second Fick's law of diffusion assuming that molecules emanate from a point source (Martínez San Segundo et al., 2020). Considering the secretion of ~8,000 glutamate molecules after the exocytosis of a single synaptic vesicle (Wang et al., 2019), it would be unlikely to reach a glutamate concentration >100 μM for distances greater than ~150 nm. In the central nervous system, for example in the striatum, approximately 80% of glutamatergic synapses are located <100 nm from the nearest astrocytic membrane (Octeau et al., 2018). This characteristic distance is typically increased in culture, thus supporting our imaging preferentially revealed the location of synapses adopting a near-physiological spatial relationship to astrocytes.

Neurons were subjected to a protocol that consisted in the application of paired pulses at an interval of 100 ms followed by a train of 6 to 20 stimuli delivered at 20 Hz. Figure 2A shows the average changes in SF-iGluSnFR.S72A fluorescence that were synchronously elicited by stimuli applied to a given neuron. Discrete dots measuring ~1 μm diameter became evident. If SF-iGluSnFR.S72A puncta were revealing the local diffusion of glutamate, they should resemble the location of synaptic contacts detected morphologically (Figure 2B). SF-iGluSnFR.S72A dots were found at a density 3.3 ± 0.2 μm^−2^, (mean ± s.e.m., *n* = 9), which was similar to the density of 3 ± 0.1 synapses·μm^−2^, (mean ± s.e.m., *n* = 10, *p* = 0.47, unpaired Student's *t*-test) found in neuronal regions where the concentration of autapses was maximal (Figure 2C). There was, however, the possibility that SF-iGluSnFR.S72A puncta were actually reflecting the contribution of groups of boutons since the high affinity variant SF-Venus-iGluSnFR.A184S shows signal correlations spanning 3.6 μm in the visual cortex (Kazemipour et al., 2019). If individual puncta displayed a population behavior, the responses obtained within a circular area of 5 μm radius should be similar. Neurotransmitter release probability assayed by paired pulse stimuli in 6 different ROIs measuring 1.3 × 1.3 μm (Figures 2A, D) was different: ROIs #1, #2 and #4 facilitated, while ROIs #3, #5 and #6 depressed in a single trial. The average paired pulse ratio of the six ROIs was 0.91, comparable to the value of 0.94 found electrophysiologically for the applied stimulation protocol (Figures 2A, D). This finding shows the capacity of a low-affinity glutamate sensor to locate release sites, thus improving the performance of high affinity variants such as SF-Venus-iGluSnFR.A184S (Aggarwal et al., 2022). We assumed that most SF-iGluSnFR.S72A puncta revealed the localization of single synaptic contacts found in close apposition to the astrocyte membrane. Autaptic cultures could maximize the presence of large boutons with enhanced neurotransmitter release compared to conventional hippocampal synapses (Liu et al., 2013) and therefore, define an experimental configuration that facilitated the identification of release sites.

**Figure 3:**
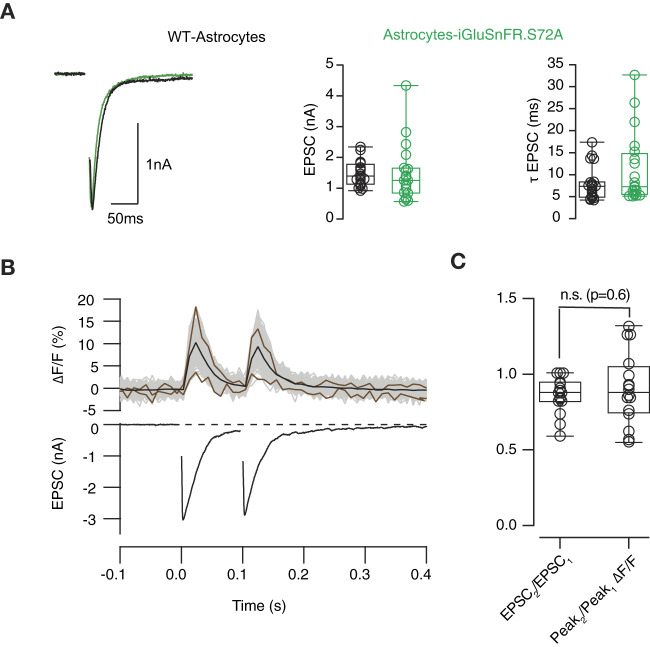
Relationship between optical and electrophysiological responses. **(A)** Left, examples of excitatory postsynaptic currents (EPSCs) obtained from autaptic hippocampal synapses grown on a layer of wild-type astrocytes (black) and astrocytes expressing SF-iGluSnFR.S72A (green). The amplitude and decay time constant of EPSCs (τ_EPSC_) were comparable in both experimental conditions. **(B)** Example showing the simultaneous recording of changes in SF-iGluSnFR.S72A fluorescence (up) and EPSCs (down) in response to two stimuli delivered at a time interval of 100 ms. The responses of individual fluorescent puncta analyzed (*n* = 48) are indicated by gray traces and the average is shown in black. Brown traces show the smallest and largest computed fluorescence responses to illustrate variability. **(C)** Comparison between paired pulse ratio (PPR) calculated from EPSCs and SF-iGluSnFR.S72A fluorescent responses. Box plots show the median (horizontal line), 25 to 75% quartiles (boxes), and ranges (whiskers) of EPSC values. Statistical differences between PPR measured electrophysiologically and optically (*n* = 18) were established using unpaired Student's *t*-test. n.s., non significant.

We aimed to confirm the association of SF-iGluSnFR.S72A fluorescence responses to electrophysiological data. Expression of SF-iGluSnFR.S72A in astrocytes did not modify postsynaptic responses (Figure 3A). Neither the amplitude nor the decay time constant of EPSCs was affected, thus discarding a possible buffering of extracellular glutamate driven by the expression of the sensor. Figure 3B shows EPSCs triggered by a pair of stimuli and the associated changes in SF-iGluSnFR.S72A fluorescence. The mean fluorescent transient was obtained by averaging the responses of all ROIs identified. There was a noticeable variability in individual responses, since they could differ in amplitude more than 4-fold. In the illustrated example, the relationship between the mean second and first fluorescent responses was of 0.88, almost identical to the ratio of 0.87 found between the second and first EPSCs. On average, optical, and electrophysiological measurements were comparable in all neurons investigated (Figure 3C, *n* = 18). Figure 3B also shows that the exponential decay of the average SF-iGluSnFR.S72A responses matched the time course of the associated EPSCs. All recordings displayed time constants of ~20 ms, thus supporting transients measured in the identified population of puncta provided an accurate temporal readout of glutamate release occurring in autaptic contacts.

**Figure 4:**
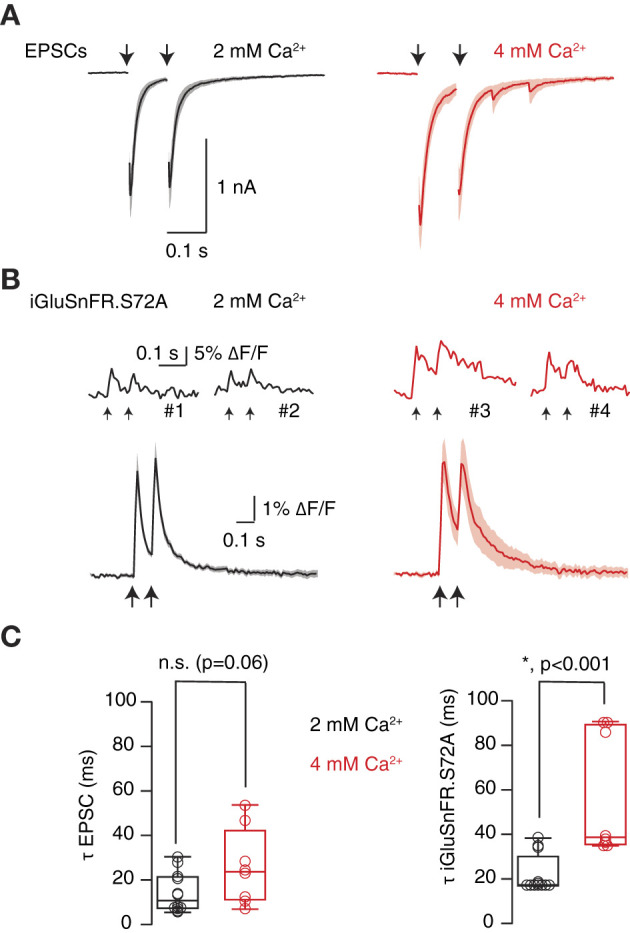
Optical detection of an increase in neurotransmitter release probability. **(A)** Enhancement of paired pulse depression upon increase from 2 mM [Ca^2+^]_e_ to 4 mM [Ca^2+^]_e_ (*n* = 8). Solid lines indicate mean excitatory postsynaptic currents (EPSCs) and shadowed areas s.e.m. **(B)** Top, examples of transient fluorescence increases obtained in four different ROIs in the indicated conditions. Down, mean SF-iGluSnFR.S72A fluorescent responses associated to the EPSCs shown in **(A)**. Traces were obtained by averaging transient increases obtained in 2 mM [Ca^2+^]_e_ and 4 mM [Ca^2+^]_e_ (*n* = 8 cells). Solid lines and shadowed areas indicate mean ± s.e.m. **(C)** The decay time of EPSCs and SF-iGluSnFR.S72A fluorescent responses was well described by a single exponential. Time constants (τ) were obtained from fits to EPSCs and SF-iGluSnFR.S72A fluorescent transients generated in response to the first stimulus of the protocol. Box plots show the median (horizontal line), 25 to 75% quartiles (boxes), and ranges (whiskers) of EPSCs (left) and fluorescent responses (right). Comparisons between fits obtained in 2 mM [Ca^2+^]_e_ and 4 mM [Ca^2+^]_e_ were established using paired Student's *t*-test (*n* = 8).

The ability of SF-iGluSnFR.S72A to detect changes in synaptic transmission was evidenced by increasing [Ca^2+^]_e_ from 2 to 4 mM. The consequent enhancement of neurotransmitter release probability moderately affected to synaptic strength (Figures 4A, B), which increased from 1.6 ± 0.2 nA to 1.8 ± 0.2 nA (*n* = 8). Paired pulse depression changed both in electrophysiological and optical measurements, showing comparable values in 4 mM [Ca^2+^]_e_ of 0.73 ± 0.1 and 0.66 ± 0.1, respectively (*n* = 8, paired Student *t*-test, *p* = 0.27, Figures 4A, B). However, the most evident effect was a lingering of EPSCs that was particularly well detected by SF-iGluSnFR.S72A (Figure 4C). The average decay time constant of EPSCs (τ_EPSC_) obtained in 2 mM [Ca^2+^]_e_ was of 14 ms, not significantly different from the associated τ_SF − iGluSnFR.S72A_ of 22 ms (paired Student's *t*-test, *p* = 0.1). The change to 4 mM [Ca^2+^]_e_ prolonged both τ_EPSC_ and τ_SF − iGluSnFR.S72A_ to 25 ms (unpaired Student's *t*-test, *p* = 0.06) and 56 ms (unpaired Student's *t*-test, *p* = 0.0006). These results could reflect an enhanced spreading of glutamate and highlight the sensitivity of SF-iGluSnFR.S72A to detect increases in neurotransmitter release probability conditions when expressed in astrocytes. We next aimed to resolve the presence of UVR and MVR in the population of autaptic contacts studied.

### Detection of univesicular and multivesicular release

**Figure 5:**
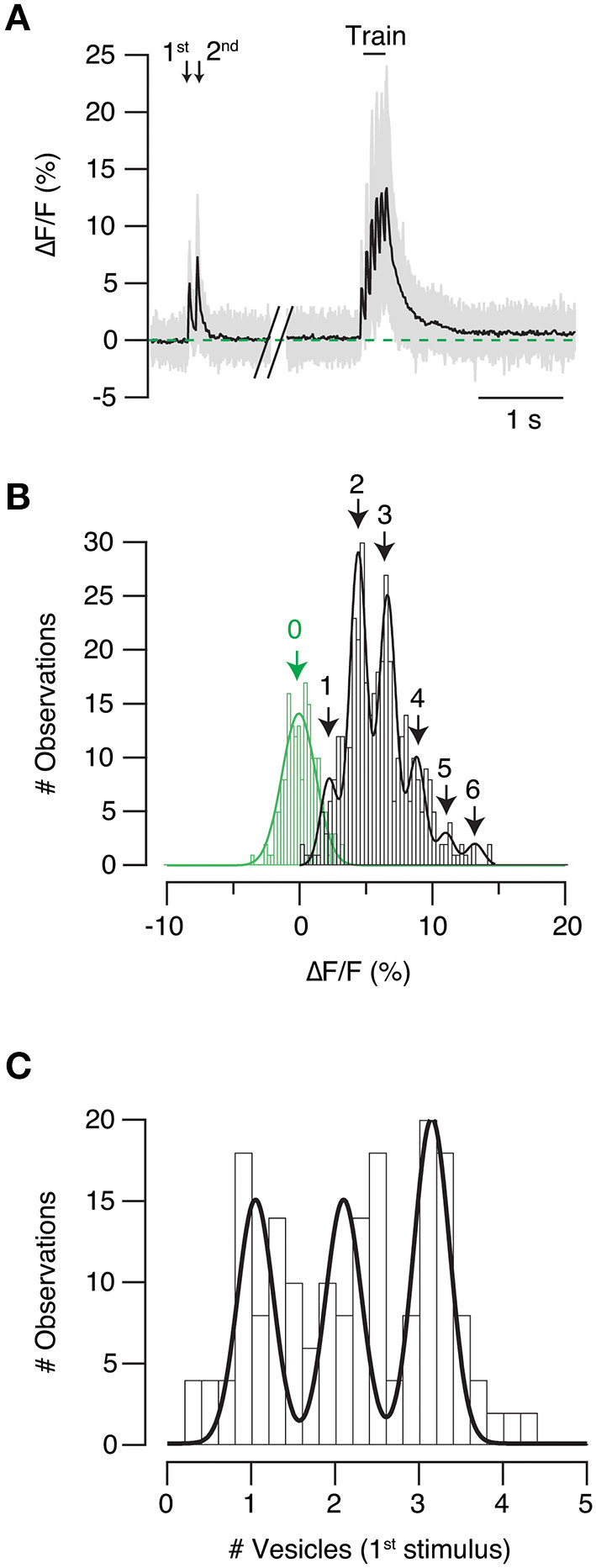
Identification of univesicular and multivesicular release. **(A)** Example showing SF-iGluSnFR.S72A fluorescent transients obtained in response to paired pulse stimulation followed by a train delivered at 20 Hz. The responses of individual puncta are shown in gray and mean is shown in black (*n* = 44). **(B)** Distribution of the amplitude of individual fluorescent responses obtained for the six stimuli of the train (black). The histogram is fitted to a gaussian mixture model (peaks 1–6) and the constant interpeak distance (2.2% ΔF/F) is considered as the quantal fluorescence response. Variations in background fluorescence measurements (green) were estimated for the selected regions of interest when the neuron was at rest. **(C)** The number of synaptic vesicles participating in neurotransmitter release in the first stimulus of the paired pulse protocol **(A)** is calculated by dividing the fluorescence of individual responses by the quantal fluorescence response. The histogram shows that presynaptic terminals studied responded to stimulation by synchronously releasing one, two or three vesicles.

SF-iGluSnFR.S72A showed a characteristic response to a train of 6–20 stimuli. The amplitude of fluorescent responses was larger at the beginning of the stimulus period and gradually decayed, which was indicative of a decrease in neurotransmitter release probability. Sensor saturation could also be contributing to the reduction of fluorescence responses, however, it would always be smaller than in high affinity variants. iGluSnFR linearly responds up to ten action potentials (Marvin et al., 2013) and has a >20 times higher affinity for glutamate than SF-iGluSnFR.S72A (Marvin et al., 2018), which makes saturation unlikely. There were, however, 3- and 4-fold differences in the fluorescence increases experimented by individual puncta in response to a single stimulus (Figure 5A gray traces, see also Figure 3B). This result evidenced the heterogenous release probability of the population of autapses investigated. Figure 5B shows an example of the distribution of fluorescence changes observed in SF-iGluSnFR.S72A puncta in response to each pulse of the stimulus train shown in Figure 5A. The goal was obtaining a readout during high and low release probability conditions occurring at the beginning and end of the train, respectively. Peaks observed were fitted to a gaussian mixture model. Populations were regularly spaced by 2.2% increases in ΔF/F. The constant distance found among peaks resembled observations obtained in *in vitro* and *in vivo* experiments using iGluSnFR (James et al., 2019; Farsi et al., 2021). Interpeak distance was thus considered as the response generated by the release of a single vesicle and was used as a calibration value. As an example, individual ΔF/F values obtained in the first stimulus of the paired pulse (Figure 5A), were divided by quantal fluorescence, and plotted in Figure 5C. The histogram was fitted to a gaussian mixture model. Three peaks were observed, indicating the release of one (UVR), 2 and 3 vesicles (MVR). This example demonstrates how different presynaptic terminals present along a single axon showed variable modalities of neurotransmitter release upon the arrival of a single action potential.

**Figure 6:**
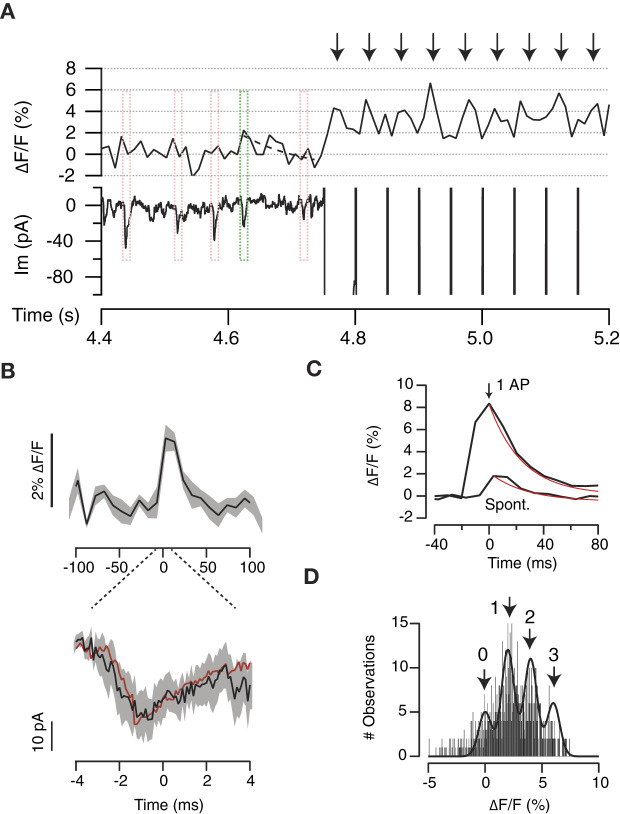
Optical detection of spontaneous neurotransmitter release. **(A)** Example showing the relationship between the changes in iGluSnFR.S72A fluorescence in a single ROI and miniature excitatory postsynaptic currents (mEPSCs). Events where the mEPSC peak coincided with a ΔF/F increase ≥2% were selected for analysis (green box). The dotted line indicates an exponential fit (τ = 10 ms). Red boxes illustrate events that did not comply with the established criteria. Arrows show the responses to a train of stimuli delivered at 20 Hz. **(B)** Average transient increase in iGluSnFR.S72A fluorescence (up) associated to the corresponding mEPSC (down). Black lines and shadowed areas indicate mean ± s.e.m. (*n* = 9). The red trace indicates the mean mEPSC obtained by averaging all spontaneous events detected (*n* = 38). **(C)** Average increases in iGluSnFR.S72A fluorescence recorded in response to a single action potential or spontaneously (same as **B**). Both transients showed comparable time constants of ~25 ms obtained from exponential fits (red traces). **(D)** Distribution of the amplitude of individual fluorescent responses obtained for a train of stimuli. The histogram is fitted to a gaussian mixture model. The interpeak distance of 2% ΔF/F is comparable to the amplitude of fluorescence changes associated to mEPSCs (see **B, C**).

To validate the use of the interpeak distance obtained in the gaussian mixture model as the quantal fluorescence value (Figure 5B), we associated changes in SF-iGluSnFR.S72A fluorescence to spontaneous neurotransmitter release. ROIs responding to stimulation were inspected for spontaneous ~2% increases in ΔF/F that occurred concomitantly to miniature EPSCs (mEPSCs, Figure 6A). Only those events where the peak of the mEPSC coincided with a ≥2% increase in ΔF/F were selected. It was not possible to extract information from individual events because they partially overlapped with background fluorescence and consequently, noise was reduced through averaging. Figure 6B shows the relationship between the mean response in SF-iGluSnFR.S72A fluorescence of 9 different mEPSCs and the average mEPSC of the recorded period (red trace, *n* = 38). The fluorescence transient obtained was thus representative of UVR because it corresponded to the exocytosis of a single vesicle. The time course of signals transients generated by a single action potential or spontaneous neurotransmitter release showed a comparable τ_SF − iGluSnFR.S72A_ of ~25 ms (Figure 6C). Differences were found only in amplitude, thus supporting that evoked neurotransmission occurred by the synchronous release of multiple vesicles. In this example most boutons supported the synchronous exocytosis of 3 or 4 vesicles (Figure 6C). The fit to a gaussian mixture model of the responses to a train of stimuli reported a 2% ΔF/F interpeak distance (Figure 6D). This value matched the increase in SF-iGluSnFR.S72A fluorescence associated to the release of a single vesicle (Figure 6B), therefore validated our experimental approach.

**Figure 7:**
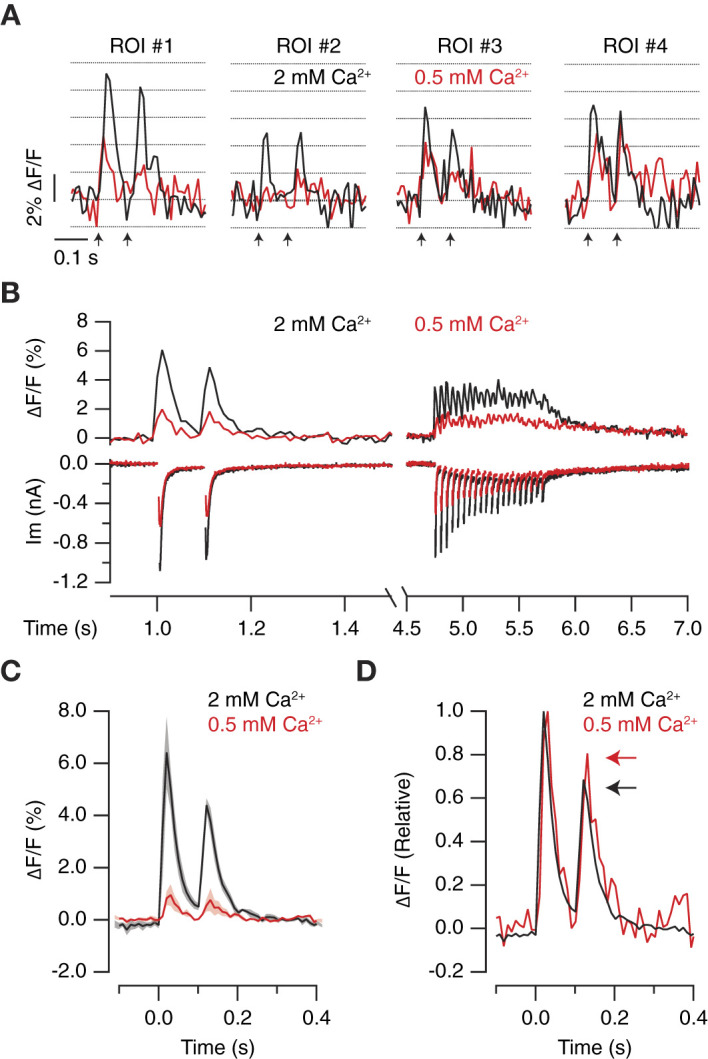
Optical quantification of the decrease in neurotransmitter release probability. **(A)** Increases in iGluSnFR.S72A fluorescence obtained in four different ROIs in 2 mM [Ca^2+^]_e_ and 0.5 mM [Ca^2+^]_e_ in response to paired pulse stimulation (arrows). Notice that the peaks of the transients obtained in a single trial approximated to multiples of ~2% increases in ΔF/F. **(B)** Simultaneous recording of postsynaptic responses and glutamate release in a neuron stimulated with two pulses at an interval of 100 ms followed by a train of 20 stimuli applied at 20 Hz. The black ΔF/F trace shows the mean response of 49 ROIs. The decrease in neurotransmitter release probability mediated by replacing 2 mM [Ca^2+^]_e_ with 0.5 mM [Ca^2+^]_e_ reduced electrophysiological and optical responses. **(C)** iGluSnFR.S72A fluorescence responses experimented a 6-fold decrease upon reducing [Ca^2+^]_e_. Solid lines and shadowed areas indicate mean ± s.e.m. (*n* = 5) neurons. **(D)** Normalized iGluSnFR.S72A fluorescence transients illustrate how the change from 2 mM [Ca^2+^]_e_ to 0.5 mM [Ca^2+^]_e_ reduces synaptic depression (arrows). Notice the time course of responses was not affected by changes in [Ca^2+^]_e_.

To supress the contribution of MVR, 2 mM [Ca^2+^]_e_ was replaced by 0.5 mM [Ca^2+^]_e_. Figure 7A shows the responses of four different ROIs in 2 mM [Ca^2+^]_e_ and 0.5 mM [Ca^2+^]_e._ Considering a quantal fluorescence change of 2% ΔF/F (see for example Figure 5B), it was possible to observe how a MVR of 2 to 4 vesicles found in 2 mM [Ca^2+^]_e_ was reduced to 0–2 vesicles in 0.5 mM [Ca^2+^]_e_. Notice how ROI#2 failed to respond to the first of the two stimuli, evidencing that our method was capable of detecting failures. The population of ROIs defined detected a decrease in neurotransmitter release probability that agreed with changes in EPSC amplitude, thus supporting a shift from MVR to UVR (Figure 7B). On average (*n* = 5), SF-iGluSnFR.S72A detected a 6-fold decrease in glutamate release (Figure 7C), which indicates that evoked neurotransmission in autaptic synapses typically involves the synchronous release of several vesicles. The decrease in neurotransmitter release probability caused a minor depression between paired pulse stimuli, which was also reported by SF-iGluSnFR.S72A (Figure 7C). This observation complements data obtained after increasing neurotransmitter release probability (Figure 4) and, makes the defined experimental configuration an ideal strategy to provide an optical readout of short-term plasticity.

**Figure 8:**
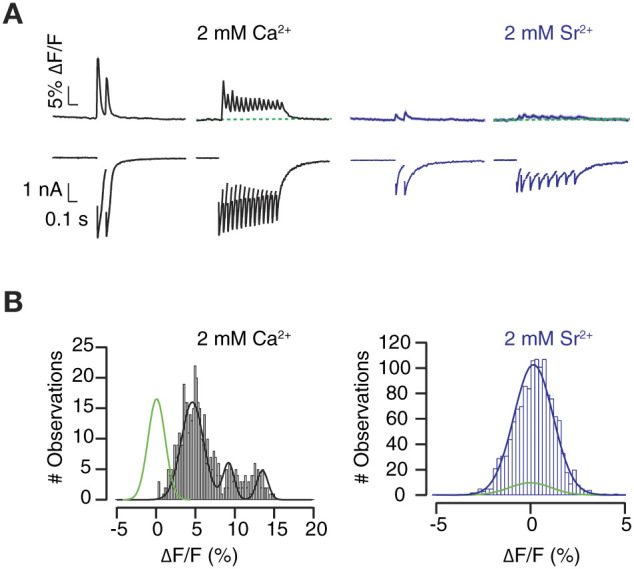
Visualization of the effect of Sr^2+^ on neurotransmitter release. **(A)** The equimolar change of 2 mM [Ca^2+^]_e_ by Sr^2+^ desynchronizes neurotransmission and suppresses SF-iGluSnFR.S72A fluorescent responses. Black and blue traces show the mean ΔF/F response (*n* = 112 ROIs) obtained in an autaptic neuron in the presence of 2 mM [Ca^2+^] and 2 mM [Sr^2+^], respectively. Shadowed areas indicate s.e.m. **(B)** Transient increases in fluorescence, fitted to a gaussian mixture model, are abolished by Sr^2+^. Notice that puncta that respond to stimulation in 2 mM Ca^2+^ fail to show fluorescence increases above background levels (gaussian fit, green) in the presence of Sr^2+^.

Changes in [Ca^2+^]_e_ affect to neurotransmitter release probability but do not alter the timing of synaptic vesicle exocytosis. We sought to desynchronize neurotransmitter release by equimolarly substituting [Ca^2+^]_e_ with Sr^2+^ (Dodge et al., 1969; Goda and Stevens, 1994). As expected, the presence of Sr^2+^ decreased EPSC amplitude and enhanced asynchronous neurotransmitter release. Responses detected by SF-iGluSnFR.S72A were affected by equimolar substitution of Ca^2+^ with Sr^2+^, however, to a greater extent than electrophysiological recordings (Figure 8A). The analysis of the illustrated example, which showed strong synaptic depression, denotes how fluorescence increases detected in the presence of 2 mM [Ca^2+^]_e_ were actually abolished by Sr^2+^ substitution (Figure 8B). Failure to detect Sr^2+^ mediated glutamate release in the selected ROIs contrasted to the capacity of SF-iGluSnFR.S72A to faithfully report changes in neurotransmitter release probability mediated by variations in [Ca^2+^]_e_ (Figures 4, 7).

Alterations in the location of neurotransmitter release sites can be poorly determined by the high affinity variant SF-Venus-iGluSnFR.A184S (Kazemipour et al., 2019), which makes SF-iGluSnFR.S72A particularly appealing to detect spatial changes in neurotransmission. An increase of the diffusion distance from the exocytic locations to the sensor could explain results obtained if synchronous and asynchronous release occurred spatially segregated. This possibility is consistent with an alteration of exo/endocytic sites upon Sr^2+^ substitution (Li et al., 2017). The nature of a possible spatial reorganization mediated by Sr^2+^ is, however unresolved. It could occur within a given bouton, i.e., by a translocation of vesicle exocytosis from synaptic to extrasynaptic sites, or involve different boutons, i.e., by inducing a shift of presynaptic terminals showing synchronous neurotransmitter release to other terminals showing asynchronous release.

### Relationship of multivesicular release to short-term synaptic plasticity

**Figure 9:**
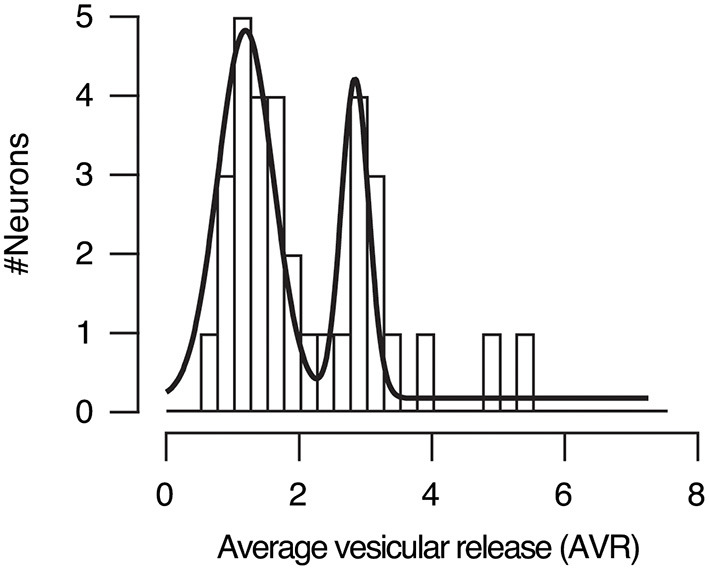
Distribution of the average vesicular release (AVR) in neurons studied (*n* = 33). The AVR refers to the average number of vesicles released by a single stimulus in the identified SF-iGluSnFR.S72A puncta. Gaussian fits defined two populations of neurons. Approximately 50% of autaptic neurons showed a preference for univesicular release (UVR, mean AVR = 1.2), while the remaining 50% displayed a predominance of multivesicular release (MVR, mean AVR = 2.8).

We assessed the prevalence of UVR or MVR in presynaptic terminals emanating from a single axon by calculating the average vesicular release (AVR). It provided an estimate of the mean number of vesicles released by a single stimulus in the population of SF-iGluSnFR.S72A puncta studied. For example, in the first stimulus of the paired pulse protocol of the neuron shown in Figure 5, the AVR was of 2.20, indicating an overall presence of MVR. If the AVR value were closer to 1, it would instead indicate that most boutons used UVR. The distribution of AVR values for all neurons studied (*n* = 33) was described by two gaussian populations with median values of 1.2 and 2.8, which represents synapses with a prevalence of UVR and MVR, respectively. Therefore, the probability of finding a neuron containing presynaptic terminals preferentially using UVR or MVR was ~0.5 (Figure 9), which agrees with morphological observations found in cultured hippocampal neurons using the ‘zap-and-freeze' method (Kusick et al., 2020). The estimated average simultaneous release of ~3 vesicles might be an overestimate due to the presence of high order events that could reflect the contribution of more than one bouton. However, it should be noticed that our value fits with the ultrastructural analysis of (Kusick et al., 2020) showing MVR driven by the fusion of 3 and 4 vesicles in 2 mM [Ca^2+^]_e_.

**Figure 10:**
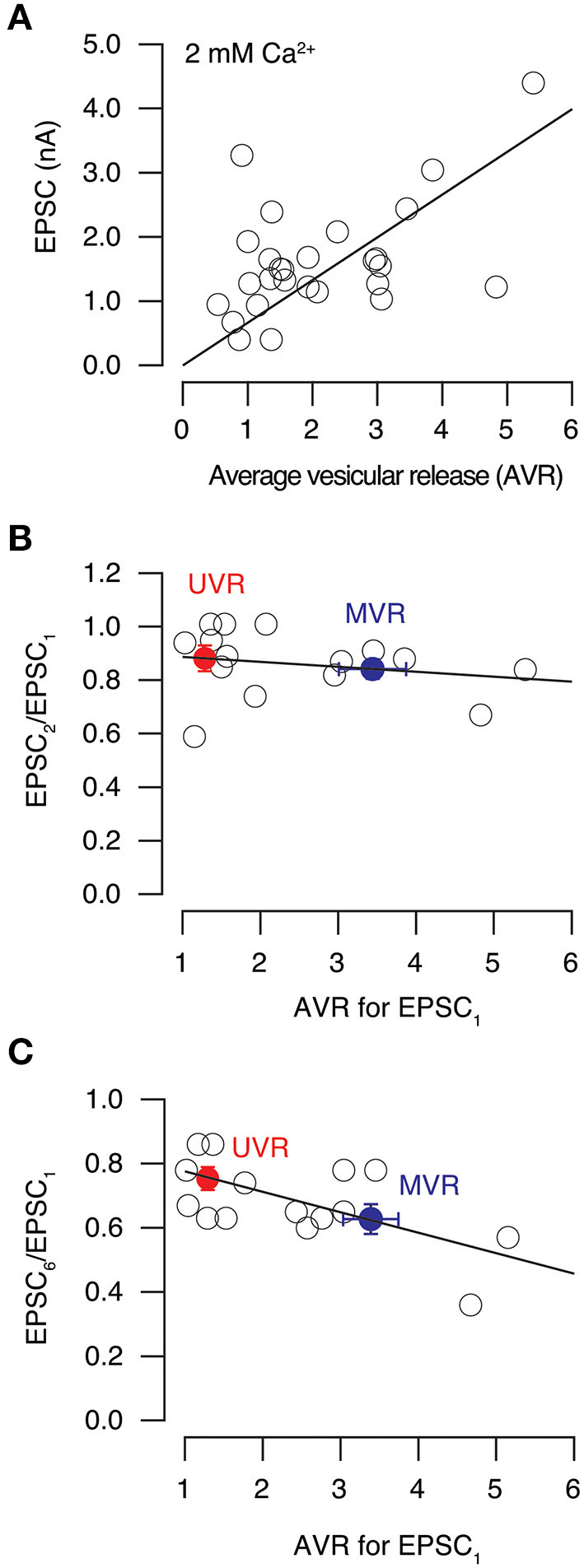
Effect of univesicular and multivesicular release on short-term synaptic plasticity. **(A)** Relationship between EPSC amplitude and average vesicular release (AVR) in neurons studied in 2 mM [Ca^2+^]_e_. Line indicates a linear fit (correlation coefficient = 0.51) to values obtained for individual cells (circles, *n* = 28). **(B)** Relationship between paired pulse ratio obtained for pulses delivered at 100 ms interval and average vesicular release (AVR) in 2 mM [Ca^2+^]_e_ (*n* = 15). The linear fit (correlation coefficient = −0.21) shows the lack of a relationship. Bins (mean ± s.e.m.) show values of neurons with a preference for univesicular release (UVR, red) or multivesicular release (MVR, blue). **(C)** Relationship between the amplitude of the 6^th^ and the 1^st^ EPSCs obtained in response to a stimulus train delivered at 20 Hz and the AVR. Circles show values obtained for individual neurons in 2 mM [Ca^2+^]_e_ (*n* = 15). Data were well described by a linear relationship (correlation coefficient of−0.64). Bins (mean ± s.e.m.) show values of neurons denoting a preference for univesicular release (UVR, red) or multivesicular release (MVR, blue).

Synaptic strength, measured as the amplitude of EPSCs in the presence of 2 mM extracellular Ca^2+^, was linearly related to the AVR (Figure 10A, correlation coefficient = 0.51). Consequently, neurons with most boutons using MVR displayed a greater synaptic potency than those neurons showing a prevalence of UVR. We next tested the relevance to short-term plasticity evoked in paired pulses delivered at an interval of 100 ms. Here, the AVR determined for the first pulse was not relevant for the degree of depression observed, which was always ~0.8 in the presence of 2 mM extracellular Ca^2+^(Figure 10B).

Since hippocampal neurons typically fire bursts of action potentials (Komendantov et al., 2019), we next evaluated the relation of AVR to short term plasticity observed in a more physiological stimulation, i.e., during trains of stimuli delivered at 20 Hz. The depression identified for the relationship EPSC_6_/EPSC_1_ ranged from 0.3 to 0.9 and was related to the AVR (linear correlation coefficient = 0.64) found for the first stimulus of the train (Figure 10C). On average, neurons with a predominance of MVR showed a 20% increase in depression compared to those displaying UVR. The prevalence of a release mode was thus useful to predict the magnitude of short-term depression evoked by sustained stimulation.

## Discussion

SF-iGluSnFR.S72A expression in the astrocytic feeder layer of autaptic hippocampal neuron cultures allows the optical tracking of synaptic transmission. Key advantages of the experimental approach are: (i) the association of electrophysiology and imaging avoiding overexpression artifacts in neurons, (ii) obtaining a fluorescent readout of the time-course of glutamate release, (iii) the detection of UVR or MVR and (iv) the identification of spatial variations in neurotransmitter release sites. Our methodology is particularly appealing to study synaptic remodeling since the sensor is not expressed in neurons. The stable presence of SF-iGluSnFR.S72A in the astrocyte membrane might be advantageous to detect morphological rearrangements of synapses. This is an aspect that has not been addressed in the current work, for example by inducing alterations in the neuronal cytoskeleton, and can be investigated in future studies. The results obtained, e.g., by altering [Ca^2+^]_e_, (Figures 4, 7, 8), suggest the validity of the approach to detect the acute retraction or elimination of synaptic contacts, which will result in a decay of iGluSnFR.S72A responses and/or disappearance of fluorescence puncta. However, obtaining a continous evaluation of synaptic positions over a long-time window is not feasible with the current methodology, which is designed for a short experimental time, i.e., 5–20 minutes. Therefore, it would not be possible to detect morphological rearrangements associated to LTP or LTD.

Previous works already showed the capacity of high affinity variants of iGluSnFR to monitor changes in the extracellular levels of glutamate *in vitro* (Marvin et al., 2013) and in intact tissue (Rosa et al., 2015), when expressed in glia. The competition with membrane transporters (Armbruster et al., 2020) and the inaccuracy to locate glutamate sources (Aggarwal et al., 2022) are, however, two important limitations that highlight the importance of using low affinity variants. Since sensors such as SF-iGluSnFR.S72A can only detect high glutamate concentrations, the gap to sites of synaptic vesicle exocytosis must be minimal. Many synapses of the central nervous system are separated by < 10 nm from astrocytes (Rollenhagen and Lübke, 2006; Octeau et al., 2018), which is a distance compatible with the use of low affinity variants of iGluSnFR. Considering that the separation of glial and synaptic membranes is increased in culture conditions, our results support that the astrocytic expression of SF-iGluSnFR.S72A is an excellent tool to obtain a physiological readout of neurotransmission. Autaptic cultures might be particularly well-suited to the current experimental approach since boutons forming autaptic synapses show an enhanced neurotransmitter release compared to conventional hippocampal synapses (Liu et al., 2013). The possibility that we were looking at a non-representative population of autapses is unlikely because data provided by optical and electrophysiological readouts were comparable. Disparities between both signals only appeared upon substitution of [Ca^2+^]_e_ by Sr^2+^, which could be induced by a spatial alteration in release sites (Li et al., 2017) or incomplete/impaired synaptic vesicle fusion (Dürst et al., 2022). The capacity of SF-iGluSnFR.S72A to optically detect changes in short-term synaptic plasticity at high frequencies is particularly appealing since it could be of great value in intact tissue.

The present work shows that presynaptic terminals found along the axon of a hippocampal neuron prefer MVR or UVR. These results confirm the presence of MVR in autapses (Ikeda et al., 2008) and agree with previous studies describing their heterogeneous neurotransmitter release probability (Murthy et al., 1997). A key aspect of the study is the demonstration that the balance between terminals showing UVR and MVR is determinant of synaptic strength. Our estimation of the precise number of vesicles supporting MVR might be affected by certain parameters that were not experimentally controlled, such as out-of-plane fluorescence, however, it should be noticed the remarkable similarity between found values and those obtained by ultrastructural observations using the “zap-and-freeze” methodology (Kusick et al., 2020). The linear relationship found between EPSC amplitude and AVR (Figure 10A) suggests that synapses can adjust their potency by adding or removing synaptic vesicles from existing release sites. We found that summation of a single synaptic vesicle to synchronous release, i.e., an increase in AVR from 1 to 2, drives a linear increment of 0.7 nA in EPSC amplitude. Considering a quantal size of 20 pA, the modification would involve 35 independent release sites, which potentially represent a ~10% of all release sites available (Ikeda and Bekkers, 2009). Neurons could thus change synaptic output by balancing UVR and MVR in a subset of their presynaptic terminals. This goal could be achieved by reorganizing the molecular structure of the active zone (Maschi and Klyachko, 2020).

The equilibrium between UVR and MVR could have important implications to set basal neurotransmission. Autaptic neuronal cultures compensate for decreases in synapse loss by activating mechanisms of presynaptic homeostatic plasticity, which involve rapid synapse formation and presynaptic potentiation (Velasco and Llobet, 2020). A selective enhancement of MVR could contribute to potentiation and act together with other pre or postsynaptic mechanisms to set baseline synaptic transmission. The ability of a given neuron to alter the balance between UVR and MVR could thus represent an efficient regulatory mechanism of synaptic strength without modifying the number of neurotransmitter release sites. Although the molecular mechanisms regulating such balance are unknown, the contribution of munc13-1 would be presumably key. This protein is present in any presynaptic terminal and plays an important role in synaptic vesicle priming (Lipstein et al., 2021). Excitatory synapses with similar morphologies express different levels of munc13-1, which could have profound consequences in the balance between terminals showing UVR or MVR (Karlocai et al., 2021). By lowering or increasing the expression of certain presynaptic proteins such as munc13-1, neurons could modify their synaptic strength.

A shift from UVR to MVR would cause an increase in short-term depression and consequently affect to the transfer of information. The synchronous exocytosis of synaptic vesicles can explain the faster development of synaptic depression because of a more efficient depletion of the readily releasable pool of synaptic vesicles (RRP). Considering the size of the RRP is finite and contains ~10 vesicles (Schikorski and Stevens, 1997), the simultaneous release of just one or 2–4 vesicles by a single action potential would be comparable. A minimal depression would thus be achieved either if UVR or MVR are the preferred modes (Figure 10B). The consequences of a prevalence of MVR would become obvious during sustained stimulation, upon a significant depletion of the RRP. Hippocampal neurons normally respond to brief trains of action potentials (Komendantov et al., 2019) and evidence shows that the rhythmicity of stimulus patterns is determinant of short-term synaptic plasticity (Vandael et al., 2020). The ability of a neuron to shift the proportion of synapses using UVR or MVR could thus predetermine its gain and low pass filtering properties.

## Data availability statement

The raw data supporting the conclusions of this article will be made available by the authors, without undue reservation.

## Ethics statement

The animal study was reviewed and approved by Generalitat de Catalunya, project #9874.

## Author contributions

PM performed research (cell culture, electrophysiology, and imaging) and analyzed data. BT performed research (molecular biology). AL designed research and wrote the paper. All authors contributed to the article and approved the submitted version.
